# Lifespan Changes in the Countermanding Performance of Young and Middle Aged Adult Rats

**DOI:** 10.3389/fnagi.2016.00190

**Published:** 2016-08-09

**Authors:** Jonathan Beuk, Richard J. Beninger, Martin Paré

**Affiliations:** ^1^Centre for Neuroscience Studies, Queen’s UniversityKingston, ON, Canada; ^2^Department of Psychology, Queen’s UniversityKingston, ON, Canada; ^3^Department of Biomedical and Molecular Sciences, Queen’s UniversityKingston, ON, Canada

**Keywords:** rats, behavioral inhibition, aging, stop signal task, lifespan, response adjustments, SSRT, post-error slowing

## Abstract

Inhibitory control can be investigated with the countermanding task, which requires subjects to make a response to a go signal and cancel that response when a stop signal is presented occasionally. Adult humans performing the countermanding task typically exhibit impaired response time (RT), stop signal response time (SSRT) and response accuracy as they get older, but little change in post-error slowing. Rodent models of the countermanding paradigm have been developed recently, yet none have directly examined age-related changes in performance throughout the lifespan. Male Wistar rats (*N* = 16) were trained to respond to a visual stimulus (go signal) by pressing a lever directly below an illuminated light for food reward, but to countermand the lever press subsequent to a tone (stop signal) that was presented occasionally (25% of trials) at a variable delay. Subjects were tested in 1 h sessions at approximately 7 and 12 months of age with intermittent training in between. Rats demonstrated longer go trial RT, a higher proportion of go trial errors and performed less total trials at 12, compared to 7 months of age. Consistent SSRT and post-error slowing were observed for rats at both ages. These results suggest that the countermanding performance of rats does vary throughout the lifespan, in a manner similar to humans, suggesting that rodents may provide a suitable model for behavioral impairment related to normal aging. These findings also highlight the importance of indicating the age at which rodents are tested in countermanding investigations.

## Introduction

Performance in a variety of tasks changes throughout the normal human lifespan. Children make rapid improvements on tests of cognitive ability into early adulthood (Casey et al., 2000), whereas normal aging is associated with impaired cognitive function, particularly inhibitory control (Hasher and Zacks, 1988; Dempster, 1992). Impaired inhibition in old age has been suggested to result from deleterious alterations in the structure, function and plasticity of cortical synapses, including reductions in total brain volume, gray matter and white matter tract integrity, most notably in frontal cortex (Courchesne et al., 2000; Raz and Rodrigue, 2006). With the recent increases in human life expectancy and inevitability of aging throughout the lifespan, it is crucial to understand the specific impairments in age-related inhibitory decline in order to elucidate the underlying etiology and design potential treatments (Morterá and Herculano-Houzel, 2012).

Rats exhibit progressive decline in the numbers of neurons in cortex, particularly in the dorsal prefrontal region, hippocampus and cerebellum from 5 to 12 months of age, with significant, although substantially variable losses apparent at 12 and 22 compared to 3 months of age (Morterá and Herculano-Houzel, 2012; Stranahan et al., 2012). Significant decreases in total synaptic and spine density, including reduced dendritic branching and plasticity have also been discovered in rat cortex with advancing age (Adams and Jones, 1982; Itzev et al., 2001; Grill and Riddle, 2002; Markham and Juraska, 2002; Bloss et al., 2011; Morrison and Baxter, 2012).

The countermanding (or stop) task requires cancelation of a prepotent response following a stop signal and has become a valuable tool for examination of inhibitory control (Lappin and Eriksen, 1966; Logan, 1994). The task allows precise estimation of response time (RT), as well as estimation of stop signal response time (SSRT), the amount of time required to cancel the primary response on ‘Stop’ trials, which is permitted by Logan and Cowan’s (1984) horse-race model of countermanding task performance that was recently validated for rats (Beuk et al., 2014). The application of rodent countermanding to examine neural correlates of inhibitory control has rapidly progressed (see Feola et al., 2000; Eagle and Robbins, 2003a,b; Van den Bergh et al., 2006; Eagle et al., 2007, 2009, 2011; Pattij et al., 2007, 2009; Robinson et al., 2008, 2009; Bari et al., 2009, 2011, 2014; Kirshenbaum et al., 2011; Bryden et al., 2012; Torregrossa et al., 2012; Bari and Robbins, 2013; Schmidt et al., 2013; Walker and Kissler, 2013; Mayse et al., 2014).

Humans performing the countermanding task exhibit shortening of RT and SSRT from childhood into young adulthood (Schachar and Logan, 1990; Williams et al., 1999; Bedard et al., 2002; Van de Laar et al., 2011). While RT for elderly, compared to younger adults has been reported as longer, inhibitory control has generally been reported as diminished (Kramer et al., 1994; Bedard et al., 2002; Andrés et al., 2008; Hu et al., 2012; Sebastian et al., 2013) although not always significantly (Williams et al., 1999; Kray et al., 2009; Van de Laar et al., 2011), possibly owing to substantial variability in SSRT lengthening with normal aging (Coxon et al., 2012; Colzato et al., 2013). RT and SSRT lengthening with advancing age may be evident in rat models; however, no studies to date have directly considered the effect of aging on countermanding task performance in rats.

Children, compared to young adults, have demonstrated significantly less go trial accuracy when performing more complex selective countermanding tasks (Bedard et al., 2002; Van de Laar et al., 2011), but not simple stop tasks (Schachar and Logan, 1990; Williams et al., 1999), whereas adults have demonstrated reduced (Hu et al., 2012; Sebastian et al., 2013) or non-differing (Williams et al., 1999; Bedard et al., 2002; Andrés et al., 2008; Kray et al., 2009) accuracy with advancing age (but see Kramer et al., 1994). Thus, inconsistencies in reported aging-related behavioral accuracy deserve further exploration, particularly with rats, which may allow more in-depth investigation of the underlying mechanisms mediating task accuracy.

The countermanding paradigm enables investigation of performance monitoring, the trial-to-trial adjustments in RT that subjects make, typically reported in humans as go trial RT shortening following consecutive go trials and lengthening following stop trials (Rieger and Gauggel, 1999; Cabel et al., 2000; Kornylo et al., 2003; Emeric et al., 2007; Li et al., 2008). Children have demonstrated RT lengthening following non-canceled stop trials that was reduced into adolescence, as well as RT lengthening following canceled stop trials that remained into young adulthood, whereas older adults exhibited reduced RT lengthening following canceled stop trials, but relatively consistent RT lengthening following non-canceled stop trials compared to young adults (Schachar et al., 2004; Van de Laar et al., 2011; Hu et al., 2012). Rats have generally demonstrated RT shortening following correct go trials and lengthening following non-canceled stop trials (Beuk et al., 2014; Mayse et al., 2014; but see Bari and Robbins, 2013); yet, changes in adaptive RT adjustments throughout the rodent lifespan have not been examined.

The countermanding paradigm has yielded valuable insights into the considerable change of inhibitory control occurring throughout the human lifespan. A validated rodent countermanding model may provide a useful tool in elucidating neurological correlates of aging-related change. Thus, we examined the performance of a cohort of rats in the countermanding task over a number of sessions when animals were approximately 7 and 12 months of age, corresponding to early- and mid-adulthood respectively (Sengupta, 2013). We found relatively unchanged SSRT between these 2 ages; however, older animals exhibited longer RT and made a higher proportion of go trial errors. While we did observe RT lengthening following non-canceled stop trials, this adjustment was consistent for rats at 7 and 12 months of age. Thus, it appears that at 12, compared to 7 months of age, rats exhibit alterations in countermanding task performance, possibly mirroring behavioral changes reported in humans with aging, providing a potential model for further examination of the neural basis of countermanding task behavioral change throughout adulthood.

## Materials and Methods

### Animals

Male Wistar rats (*N* = 16) were tested for adult lifespan changes in countermanding task performance. Rats were bred by Charles River Laboratories (St. Constant, QC, Canada) and weighed 150–200 g at the start of training, corresponding to approximately 45 days of age, based on Wistar rat growth curves (Charles River Laboratories). Repeated testing was administered to all animals at approximately 7 months of age and then again at approximately 12 months of age (see Procedure). Subjects were pair-housed in clear plastic cages (50.0 cm × 40.0 cm × 20.0 cm high) with woodchip bedding (Beta Chip; Northeastern Products Corp., Warrensberg, NY, USA) in an environmentally controlled colony room with a reversed 12-h light–dark cycle (lights off at 0700 h). Rats were given free access to water, with food (LabDiet 5001; PMI Nutrition Intl, Brentwood, MO, USA) restricted (see Procedure). All animal care and experimental protocols were approved by the Queen’s University Animal Care Committee and were in accordance with the guidelines of the Canadian Council on Animal Care and the Animals for Research Act. These animals were subsequently administered acute amphetamine, which is not reported presently, but was included in a report by Beuk et al. (2014).

### Apparatus

For a more complete description of the methods, see Beuk et al. (2014). Data were collected from four identical operant chambers (30.5 cm × 24.1 cm × 21.0 cm high; ENV-008, Med Associated, Inc., St. Albans, VT, USA). Chambers contained a clear polycarbonate door, rear wall and ceiling with a 1.0 cm separated stainless-steel parallel rod floor (0.5 cm diameter rods). On both side walls, aluminum vertical posts separated the walls into three panels. On one wall, the far panel contained a 2.8-watt incandescent house light, 1.0 cm below the ceiling and 5.0 cm above a tone generator, which emitted a tone with a chamber-specific frequency that ranged from 2400 to 3400 Hz at an intensity of 75 dB. The middle panel contained a food pellet receptacle (5.1 cm × 5.1 cm × 2 cm deep) that was 3.0 cm above the grid floor. Dustless precision food pellets (45 mg) from Bio-Serv (Frenchtown, NJ, USA; product number: F0021) were dispensed from a pedestal-mounted pellet dispenser located outside of the chamber. On the opposite wall, each of the three panels was outfitted with a 2.5 cm diameter LED stimulus light that was 4.5 cm below the ceiling and 5.0 cm above a retractable response lever (4.8 cm × 1.7 cm × 1.3 cm thick). Each chamber was isolated in a sound-attenuating case. Programming and data analysis was controlled by MED-PC^®^ IV software (Med Associated, Inc.).

### Procedure

Rats were initially housed in pairs with food and water available ad libitum. For days 3–7 of colony room habituation, rats were handled in pairs approximately 5 min/day. Food access was restricted on the 7th day to 1 h free-feeding/day for the majority of training. Food access was increased to 2 h/day later in the study to maintain weight growth.

Animals were trained to press the lever below an illuminated light-emitting diode stimulus light for sucrose pellet reward. Next, rats were trained to withhold lever press responding in the presence of a tone (acting as the stop signal) in order to obtain sucrose pellet reward. Criterion for training sessions was correct responding on ≥80% of the last 100 trials in a session.

**FIGURE 1:**
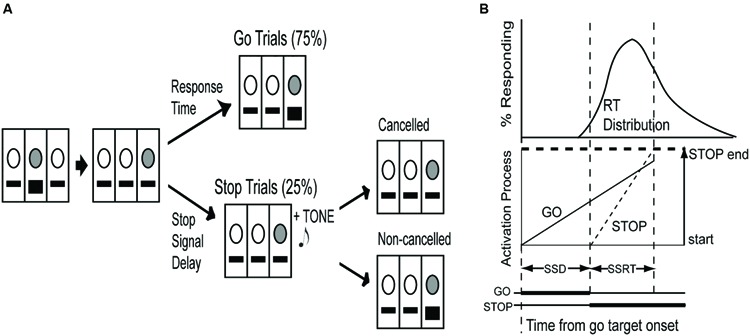
**The countermanding paradigm in rats. (A)** The light above the center lever was illuminated (indicated by gray circle) and required a center lever press (indicated by black square) to initiate a trial. Upon center lever press, a light was immediately illuminated randomly above either the left or right lever, acting as the go stimulus. On go trials (75%), pressing the lever directly below the illuminated light before the end of a time limit resulted in food reward. On stop trials (25%) an auditory tone was presented as a stop signal at varying delays from go stimulus onset and required cancelation of the lever press for food reward, whereas a non-canceled lever press resulted in a 10-s timeout interval. **(B)** The race model of countermanding performance (Logan and Cowan, 1984) proposes that two independent processes, one initiated the go signal, the other by the stop signal, race toward a threshold, whereby the first process to cross the threshold wins the race and determines the behavioral outcome. The distribution of response times (RTs) can be integrated until the integral equals the probability of canceling a response at a given stop signal delay (SSD). The time at this point minus the stop process start time (i.e., stop signal onset) can be represented as the stop signal response time (SSRT; adapted from Paré and Hanes, 2003).

Countermanding sessions (60 min) consisted of 75% go trials and 25% stop trials presented randomly (**Figure 1A**). The house light was always illuminated except during timeout intervals (see below). Initially, the light above the center lever was illuminated, requiring a center lever press to initiate a trial. Immediately following a center lever press, a target light (acting as the go signal) was randomly illuminated above either the left or right lever, signifying the lever below the illuminated light as the target lever. The target lever was only active for a time limit previously established for each individual rat in countermanding task training, which eliminated approximately the slowest 10% of the RT distribution (1.0–1.6 s). This time limit was imposed to encourage fast responding.

In go trials, rats were required to press the target lever before the end of the time limit in order to receive sucrose pellet reward. The elapsed time from center to target lever press was recorded as go RT. If the target lever was not pressed before the end of the time limit, or a non-target lever was pressed, the response was considered incorrect and resulted in a timeout interval, whereby all lights in the chamber, including the house light, were turned off for 10-s. In stop trials, a tone, acting as the stop signal, was presented concurrently with target light illumination for the length of the time limit plus an additional 300 ms. The stop signal instructed animals to inhibit a lever press to receive reward. Any lever press during stop trials was considered an incorrect response and resulted in a timeout interval, whereby all lights in the chamber, including the house light, were turned off for 10-s. A 5-s intertrial interval, where only the house light was illuminated, directly preceded the onset of the next trial following both correct and incorrect responses.

The stop signal delay (SSD) was determined with a staircase procedure (Levitt, 1971). The staircase procedure was employed to obtain successful cancelation on approximately 50% of stop trials in order to obtain accurate estimates of SSRT and account for differences in go trial RT between different animals (Band et al., 2003). Sessions initially began with a 100-ms SSD, which was adjusted throughout the session by 100-ms steps. Thus, SSD increased by 100 ms on the next stop trial if a lever press was correctly countermanded or decreased by 100 ms on the next stop trial if a non-canceled lever press was made. Finally, if a lever press on a stop trial occurred before stop signal presentation, the trial was recorded as a non-canceled response and SSD was decreased on the following stop trial by 100 ms; however the rat was given a sucrose pellet and a 5-s intertrial interval (i.e., it appeared as a go trial to the rat). Consequently, these trials were not included in RT adjustment analysis. Immediately prior to all countermanding task sessions, rats completed training blocks of 10 go trials followed by 10 stop trials with a trial time limit of 1.5 s.

Subjects were trained until they could consistently meet performance criteria (SSRT > 50 ms, > 100 total trials, < 30% errors on go trials) in countermanding task sessions (approximately 95 training sessions over 5 months). At this point, all animals were tested in the countermanding task (1 session/day; 3–5 sessions/week for a total of approximately 11 test sessions) for a 17-days period that occurred at approximately 7 months of age. Animals received intermittent task training (3–7 sessions/week for a total of approximately 72 training sessions) in the subsequent 5 months, after which all animals were tested again (1 session/day; 3–7 sessions/week for a total of approximately 13 test sessions) in the countermanding task over a 14-days period. This second testing phase occurred at approximately 12 months of age.

### Statistical Analysis

Five sessions from each age epoch for each rat were compared to study lifespan changes in countermanding task performance. Within each countermanding task session, the number of non-canceled responses made at each SSD was divided by the total number of stop trials at that SSD to calculate the inhibition function (IF). Sessions that did not exhibit an IF increase as SSD increased of at least 0.5 between the minimum and maximum value, which is considered a prerequisite of suitable countermanding task performance, were omitted from analysis (cf. Kapoor and Murthy, 2008). Sessions were also omitted from analysis if they did not meet performance criteria (see above) which would suggest that subjects did not perform the task according to instructions (e.g., Ghahremani et al., 2012). The five sessions nearest to the end of the 2 age epochs that met performance criteria were selected for further analysis. If SSD climbed more than two consecutive steps later in a 1-h countermanding task session and did not regress back toward mean session SSD, all trials following the canceled stop trial that initiated the run were excluded from analysis. Increased SSD later in sessions was associated with slower, unstable responding and can be indicative of decreased motivation and/or attention, which can lead to substantial SSRT miscalculations (Verbruggen et al., 2013). One subject was omitted from the experiment due to health complications. Five more subjects did not consistently meet performance criteria during one or both of the age epochs, which required their omission from the study. This left 10 rats for subsequent analysis of lifespan changes in countermanding performance.

We employed the integration method for estimation of SSRT, derived from Logan and Cowan’s (1984) horse-race model of stop-task performance (**Figure 1B**). First, we calculated mean SSDs from the peaks and valleys of each SSD run and the midpoint of every second SSD run and averaged these two measures to estimate the SSD where the probability of making a non-canceled response was 0.5 (Levitt, 1971). The horse-race model assumes independence of go and stop processes; therefore we integrated the distribution of go trial RTs until the integral equaled the RT at which the probability of making a non-canceled response on a stop trial was 0.5. Assuming SSRT is constant, SSRT is equal to this instant (i.e., the time at which the stop process ends) minus the SSD where the probability of making a non-canceled response was 0.5 (i.e., the instant when the stop process began).

We took into account the prospect that a correctly inhibited stop trial may actually reflect a failed go response, as rats omitted responding on a small proportion of go trials. Thus, the inhibition probability data were corrected using a procedure modified from Tannock et al. (1989): Y = (X-O)/(N-O), where for a given SSD, Y is the corrected proportion of non-canceled stop trials, X is the observed number of non-canceled stop trials, N is the total number of stop trials and O is the proportion of omissions that occurred on all go trials.

The coefficient of variation (CV), which was estimated as the ratio of the standard deviation (SD) to the mean of the RT distribution, was calculated as a measure of go trial RT variability for each session (Bellgrove et al., 2004). CVs were also calculated to determine day-to-day performance variability by comparing the means from the five sessions to their SD. We examined RT adjustments by isolating blocks of three consecutive trials where a correct go trial response occurred prior to and following a correct go trial response, a non-canceled stop trial response or a canceled stop trial response. Due to substantial variation in RT distributions among animals, this change in RT was also standardized for each individual trial sequence by computing a Z-score:

$$Z=\left[g\,o\,2\,R\,T_{\left(t\,r\,i\,a\,l\right)}-g\,o\,1\,R\,T_{\left(m\,e\,a\,n\right)}\right]/g\,o\,1\,R\,T_{\left(S\,D\right)}$$

For each rat, the RT adjustments and Z-scores from each session within a particular age epoch were combined with the other sessions from that age to determine overall means. Z scores were also computed to examine the change in go trial RT and SSRT at 7 and 12 months of age in order to account for the day-to-day variability of performance. For these data:

$$Z=\left[R\,T_{12\,\;m\,o\,n\,t\,h\,s\,\;s\,e\,s\,s\,i\,o\,n}-R\,T_{7\,\;m\,o\,n\,t\,h\,s\,\;m\,e\,a\,n}\right]/R\,T_{7\,\;m\,o\,n\,t\,h\,s\,\;S\,D}$$

Countermanding task performance variables were calculated with custom written MATLAB scripts (The MathWorks, Inc., Natick, MA, USA). Analysis of variance (ANOVA) was conducted to analyze differences in RT, SSRT and adaptive RT adjustments. Paired samples *t*-test were used to compare the average RTs of the go trial before and following each interleaved trial type as well as Z scores and differences in CV for each age epoch. Statistical analysis was conducted with SPSS software (IBM SPSS Statistics for Windows, Armonk, NY, USA). All analysis was conducted using a significance level of 0.05.

## Results

### Total Number of Trials

**FIGURE 2:**
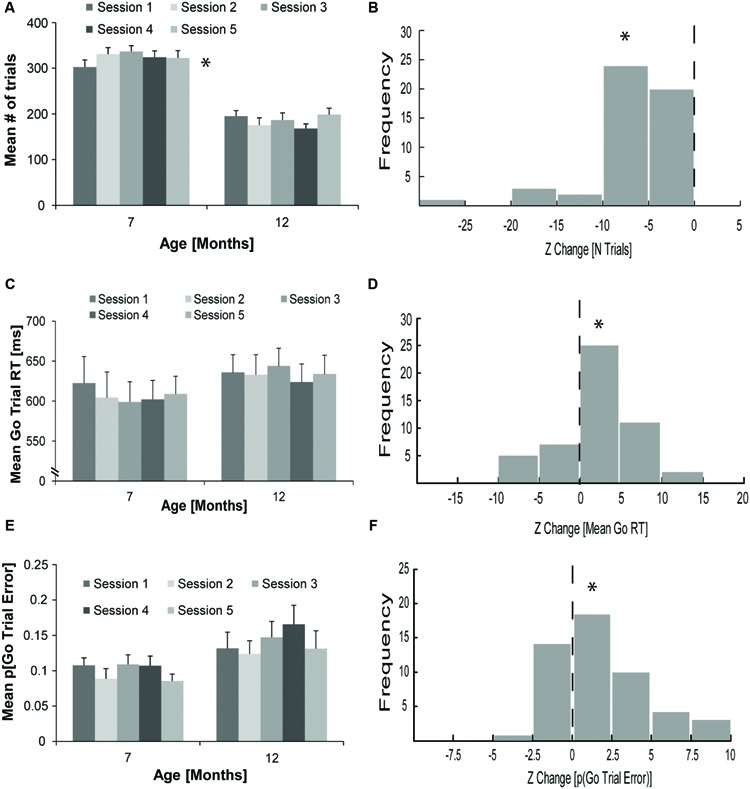
**Changes in countermanding task performance of rats with aging. (A)** Mean ± standard error of the mean (SEM) total number of trials performed in five individual, 1-h countermanding task sessions (light–dark gray bars) for rats (*N* = 10) at 7 and 12 months of age (^∗^significant main effect of age with analysis of variance [ANOVA]). **(B)** Frequency distribution (*n* = 50) for Z change in the total number of trials performed for rats (*N* = 10) comparing the total number of trials performed in five individual sessions at 12 months of age to their overall mean total number of trials performed over five sessions at 7 months of age (^∗^significant difference from zero, one-sample *t*-test). **(C)** Mean (± SEM) go trial RT in five individual countermanding task sessions (light–dark gray bars) for rats (*N* = 10) at 7 and 12 months of age. **(D)** Frequency distribution (*n* = 50) for the Z change in mean go trial RT for rats (*N* = 10) comparing mean go trial RT in five individual sessions at 12 months of age to their overall mean go trial RT over five sessions at 7 months of age (^∗^significant difference from zero, one-sample *t*-test). **(E)** Mean (± SEM) proportion of go trial errors (p[go trial error]) in five individual countermanding task sessions (light–dark gray bars) for rats (*N* = 10) at 7 and 12 months of age. **(F)** Frequency distribution (*n* = 50) for the Z change in the p(go trial error) for rats (*N* = 10) comparing the p(go trial error) in five individual sessions at 12 months of age to their overall p(go trial error) over five sessions at 7 months of age (^∗^significant difference from zero, one-sample *t*-test).

The average ± standard error of the mean (SEM) total number of trials completed by rats (*N* = 10) over all of the 1-h sessions was 323.42 ± 12.73 at 7 months of age and 184.76 ± 9.44 at 12 months of age. As displayed in **Figure 2A**, this decline in trials completed per session at 12 compared to 7 months of age was significant. A repeated measures ANOVA with age (7 or 12 months) and session (1–5) as within-subject factors revealed a significant main effect of age [*F*(1,9) = 83.70, *p* < 0.01] and a significant age and session interaction [*F*(4,36) = 2.75, *p* = 0.04]. The main effect of session was not significant [*F*(4,36) = 0.95, *p* = 0.45]. To account for variability in the number of trials performed in each of the five sessions, we calculated the CV for each rat at both 7 and 12 months of age. The mean CV in the total number of trials performed per session ± SEM was significantly higher at 12 (0.19 ± 0.01) compared to 7 (0.08 ± 0.01) months of age [*t*(9) = 5.16, *p* < 0.01]. Due to the increased variability, Z scores were calculated to assess the change in the total number of trials performed in sessions at 12 months of age compared to the mean number of trials performed in sessions at 7 months of age. As displayed in **Figure 2B**, the reduction in the total number of trials performed at 12, compared to 7 months of age (Mean Z ± SEM = -7.12 ± 0.66) was significantly different from zero [one-sample *t*-test, *t*(49) = 10.77, *p* < 0.01].

### Go Trial RT

As illustrated in **Figure 2C**, the average go trial RT (±SEM) was somewhat longer for rats at 12 (634.17 ± 22.17 ms) than 7 (607.42 ± 26.84 ms) months of age. A repeated measures ANOVA with age (7 or 12 months) and session (1–5) as within-subject factors did not reveal a significant main effect of age [*F*(1,9) = 1.91, *p* = 0.20], session [*F*(4,36) = 1.50, *p* = 0.22], or a significant interaction [*F*(4,36) = 1.15, *p* = 0.35]. Because RT lengthening at 12, compared to 7 months of age was predicted, we computed Z scores of the change in go trial RT for each 12 months session in relation to mean 7 months go trial RT for each rat to better account for RT variability. As shown in **Figure 2D**, the increase in go trial RT (Mean Z ± SEM = 2.06 ± 0.68) was significantly different from zero [one-sample *t*-test, *t*(49) = 3.05, *p* > 0.01]. We did not observe a significant difference in the mean go trial RT CV ± SEM at 7 (0.03 ± 0) or 12 (0.03 ± 0.01) months of age [*t*(9) = -0.06, *p* = 0.95]. To examine if individual session go trial RT CV differed significantly between age epochs, a repeated measures ANOVA with age (7 or 12 months) and session (1–5) as within-subject factors was conducted. No significant main effects or interactions were discovered (data not shown).

### Go Trial Accuracy

To examine whether rats committed more errors on go trials at 12 compared to 7 months of age, we conducted a repeated measures ANOVA with age (7 or 12 months) and session (1–5) as within-subject factors on the proportion of overall go trials that were incorrect lever presses and omissions. As displayed in **Figure 2E**, ANOVA revealed a near significant main effect of age [*F*(1,9) = 4.47, *p* = 0.06], and session [*F*(4,36) = 2.39, *p* = 0.07] and a non-significant interaction [*F*(4,36) = 0.44, *p* = 0.78]. To account for variability in the proportion of go trial errors, we computed a Z score for each session at 12 months of age compared to the mean at 7 months of age. The increase in the proportion of go trial errors (Mean Z ± SEM = 1.60 ± 0.38) was significantly different from zero [one-sample *t*-test, *t*(49) = 4.20, *p* < 0.01], as illustrated in **Figure 2F**. Incorrect lever presses on go trials occurred on less than 4% of all go trials for each session and accounted for less than 4% of go trial errors on average. Thus, the vast majority of go trial errors in countermanding task session consisted of errors of omission. Of note, a significant correlation was not found between Z scores accounting for the change in the proportion of go trial errors and Z scores accounting for the change in mean go trial RT (*R*^2^ < 0.01, *p* = 0.89); therefore, the observed increase in the proportion of go trial errors at 12 months of age was not likely related to the observed lengthening of go trial RT.

### Stop Signal Response Time

**FIGURE 3:**
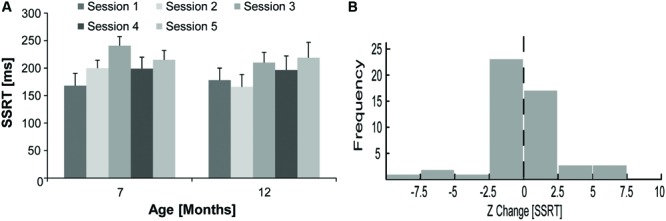
**Stop signal response time for rats at 7 and 12 months of age. (A)** Mean ± SEM SSRT in five individual countermanding task sessions (light–dark gray) for rats (*N* = 10) at 7 and 12 months of age. **(B)** Frequency distribution (*n* = 50) for the Z change in SSRT for rats (*N* = 10) comparing mean SSRT in five individual sessions at 12 months of age to their overall mean SSRT over five sessions at 7 months of age.

The overall mean SSRT ± SEM did not change substantially for rats at 12 (193.84 ± 12.99 ms) compared to 7 months of age (204.42 ± 12.37 ms). For the data displayed in **Figure 3A**, a repeated measures ANOVA with age (7 or 12 months) and session (1–5) as within-subject factors did not reveal a significant main effect of age [*F*(1,9) = 0.76, *p* = 0.41], session [*F*(4,36) = 2.33, *p* = 0.08], or a significant interaction [*F*(4,36) = 0.70, *p* = 0.60]. Z scores were calculated for the change in SSRT for each 12 months session in relation to mean 7 months SSRT for each rat to account for possible day-to-day variability in SSRT. As shown in **Figure 3B**, the distribution of Z scores for the change in SSRT at 12 compared to 7 months of age (Mean Z ± SEM = -0.04 ± 0.37) was not significantly different from zero [one-sample *t*-test, *t*(49) = -0.12, *p* = 0.91]. The Z change in SSRT was also not significantly correlated with the Z change in go trial RT (*R*^2^ < 0.02, *p* = 0.38). We compared the CV of SSRT for rats between 12 and 7 months of age to examine if there was a substantial change in the day-to-day variability of SSRT. While we did observe some increase in the mean ± SEM CV of SSRT at 12 (0.34 ± 0.03) compared to 7 (0.24 ± 0.05) months of age, this difference was not statistically significant [paired samples *t*-test, *t*(9) = 1.85, *p* = 0.10].

### Adaptive RT Adjustments

**FIGURE 4:**
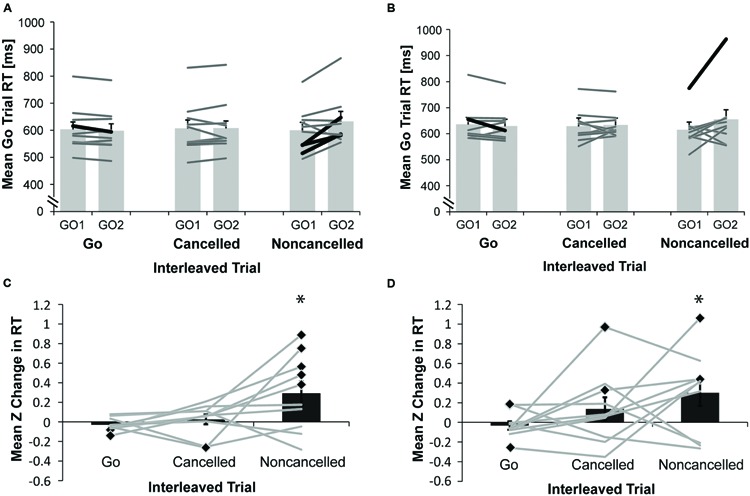
**Adaptive RT adjustments. (A,B)** Light gray bars compare mean ± SEM go trial RT for sets of three consecutive trials where go trials immediately preceded (GO1) and followed (GO2) either a correct go, a canceled stop or a non-canceled stop interleaved trial, for rats (*N* = 10) performing the countermanding task at **(A)** 7 months of age or **(B)** 12 months of age. Heavy black lines show individual animal mean RTs where GO1 and GO2 were significantly different (paired *t*-test, *p* < 0.05) whereas dark gray lines show individual animal mean RTs where GO1 and GO2 were not significantly different. **(C,D)** Dark gray bars show mean (± SEM) Z change in go trial RT for trials following, compared to trials preceding, an interleaved correct go, canceled stop or non-canceled stop trial for rats (*N* = 10) at **(C)** 7 months of age; or **(D)** 12 months of age, where Z = [GO2RT(trial) – GO1RT(mean)]/GO1RT(standard deviation). Light gray lines represent Z scores for each individual animal at each interleaved trial type (^∗^significant difference of mean Z score from zero with one-sample *t*-test, *p* < = 0.05; black diamonds indicate significant difference of an individual animals Z score from zero with a one-sample *t*-test, *p* < 0.05).

We tested if rats made adaptive RT adjustments and whether this behavior changed between 7 and 12 months of age by comparing mean RTs in correct go trials immediately prior to and following three different types of interleaved trials: (1) a correct go trial; (2) a canceled stop trial; or (3) a non-canceled stop trial. The mean ± SEM RT for each rat as well as the average RTs across all rats are shown for rats at both 7 (**Figure 4A**) and 12 months of age (**Figure 4B**). Significant RT shortening following a correct go trial was observed for one rat at both 7 and 12 months of age, while significant RT lengthening following a non-canceled stop trial was found for three rats at 7 months of age and one rat at 12 months of age (paired samples *t*-test, *p* < 0.05). Because the distribution of RTs varied substantially among rats, we standardized the change in RT as a result of each different interleaved trial type as Z scores for each rat, as displayed in **Figures 4C,D** for rats at 7 and 12 months of age respectively. A repeated measures ANOVA with age (7 or 12 months) and interleaved trial type (go, canceled, or non-canceled) as within-subject factors revealed a significant main effect of interleaved trial type [*F*(2,18) = 4.14, *p* = 0.03]. A significant main effect of age was not found [*F*(1,9) = 0.40, *p* = 0.54], nor was a significant interaction [*F*(2,18) = 0.43, *p* = 0.66]. A follow-up pairwise comparison of the significant main effect adjusted with Bonferroni correction revealed that the effect was primarily driven by a near-significant difference in mean Z scores ± SEM for correct go interleaved trials (-0.03 ± 0.03) compared to mean Z scores ± SEM for non-canceled interleaved trials (0.30 ± 0.11; *p* = 0.08). Because RT adjustments were expected, planned paired-samples *t*-test were conducted on the Z scores for each interleaved trial type. The distribution of Z scores for non-canceled interleaved trials was significantly different from zero for rats at both 7 [*t*(9) = 2.39, *p* = 0.04] and 12 months of age [*t*(9) = 2.25, *p* = 0.05]. Z scores did not differ significantly from zero when the interleaved trial was a correct go [*t*(9) = -1.36, *p* = 0.21; *t*(9) = -0.77, *p* = 0.46] or canceled stop trial [*t*(9) = 0.51, *p* = 0.63; *t*(9) = 1.16, *p* = 0.28] for rats at 7 and 12 months of age respectively.

## Discussion

At 12, compared to 7 months of age, rats performed fewer trials, exhibited longer RT and made a higher proportion of go trial errors, primarily consisting of omissions, in 1-h countermanding task sessions. Variation in SSRT was not observed at these two ages. RT lengthening following non-canceled stop trials was observed for rats at both 7 and 12 months of age. This is the first study to specifically examine changes in countermanding task performance of rats during the lifespan.

Older, compared to younger adult humans, consistently demonstrate lengthening of RT in countermanding (Kramer et al., 1994; Williams et al., 1999; Rush et al., 2006; Van de Laar et al., 2011; Coxon et al., 2012; Hu et al., 2012, 2013), Go/Change (Christ et al., 2001), Go/No-Go (Nielson et al., 2002; Sebastian et al., 2013), and flanker (Colcombe et al., 2005) tasks. RT lengthening later in the human lifespan has been proposed to result from impaired information processing speed and executive dysfunction (Salthouse, 1996; Albinet et al., 2012) or simply a preference for accuracy over speed (Ratcliff et al., 2001). Rats have exhibited longer RT at 24, compared to 6 months of age in an operant delayed response task, which was associated with reduced activity in the medial prefrontal cortex during go stimulus presentation, in support of a frontal impairment account of age-related RT lengthening in rats (Caetano et al., 2012).

Support for variation in countermanding task accuracy throughout the human lifespan has been inconclusive, with reports of impaired (Hu et al., 2012; Sebastian et al., 2013), improved (Kramer et al., 1994) or non-differing (Williams et al., 1999; Bedard et al., 2002; Andrés et al., 2008; Kray et al., 2009) error rates for older compared to younger adults. Reduced white matter tract integrity in corticostriatal tracts was associated with longer RT and increased errors for older adults performing a speeded response task (Forstmann et al., 2011). Increased errors and RT lengthening were observed in aged rats performing a simple reaction time task, suggesting that changes in latency and accuracy were dependent (Burwell and Gallagher, 1993). Our findings indicate that longer RT and decreased accuracy occurred independently for rats at the ages tested. It will be of interest to more precisely investigate the role of impaired sensory processing or executive function in the age-related impairments in accuracy and RT observed presently.

Inhibitory impairment for older adults performing the countermanding task has not been discovered in a number of experiments (Williams et al., 1999; Kray et al., 2009; Van de Laar et al., 2011). One possible explanation is that older participants with better inhibitory control were tested, as substantial variability in stopping speed has been reported in the elderly (Coxon et al., 2012; Colzato et al., 2013). These studies may have also employed simple countermanding tasks, whereas more complex tasks may impose a larger cognitive burden on the elderly, augmenting inhibitory deficits. Moreover, training effects may have masked an overall inhibitory deficit. Importantly, inhibitory impairment was generally not observed until beyond 60 years of age (Kramer et al., 1994; Bedard et al., 2002; Andrés et al., 2008; Hu et al., 2012; Sebastian et al., 2013). Presently, rats were tested up to 12–13 months of age, corresponding to mid-adulthood (Sengupta, 2013). Investigation of older animals is required to confirm whether stopping lengthens in elderly animals.

Longer SSRT and RT in elderly humans may be dependent variables, providing evidence of impaired inhibitory processing resulting in strategic RT lengthening in order to proactively increase accuracy (Andrés et al., 2008; Van de Laar et al., 2011; Anguera and Gazzaley, 2012); however, age-related changes in selective inhibitory control were reported to occur independently of overall RT lengthening (Bedard et al., 2002). The present experiment suggests that lengthening of RT occurred independently of SSRT in rats for the ages tested, contradicting the hypothesis that RT lengthening may result as a strategic by-product of impaired inhibition. Future studies should examine whether inhibition is correlated with RT lengthening in more elderly animals.

The prefrontal cortex may be particularly vulnerable to the aging process, with substantial age-related structural and functional modification reported, supporting the hypothesis that general inhibitory deficiency, resulting from prefrontal cortex deterioration, may be a universal feature of age-related decline in cognition (Hasher and Zacks, 1988; Tisserand and Jolles, 2003; Tisserand et al., 2004; Burke and Barnes, 2006; Anguera and Gazzaley, 2012). Decreased activation and white-matter tract integrity in prefrontal cortex, presupplementary motor area and basal ganglia, as well as decreased gray matter volume in prefrontal cortex and presupplementary motor area were associated with countermanding impairment in elderly adults, suggesting inhibitory deficits related to diminished recruitment of executive networks (Coxon et al., 2012, 2014; Hu et al., 2012, 2013; Sebastian et al., 2013). Moreover, older, but not younger adults carrying the C957T C/C genetic polymorphism, putatively associated with elevated striatal dopamine and dopamine D2 receptor expression (Hirvonen et al., 2009a,b), more efficiently countermanded responses than others in their age group, suggesting a genetic component of individual variation revealed with normal aging (Colzato et al., 2013). These specific neural correlates of countermanding task behavioral impairment observed in elderly humans are potentially amenable to further investigation with rodents.

Rats older than 24 months were impaired on acquisition of delayed matching as well as olfactory and visual reversal learning compared to rats 6 months of age or younger (Dunnett et al., 1988; Brushfield et al., 2008). Rats older than 18 months of age required more trials to learn reversals in a set-shifting task than younger animals (Tait et al., 2013). Moreover, rats at 16 or 21 months of age made more errors and required more sessions before reaching criteria in an 8-arm radial maze and exhibited impaired acquisition and retention of a passive avoidance conditioned response compared to 3-months-old (Granger et al., 1996; Winter, 1997). Similarly, rats older than 26, compared to 6 months of age exhibited substantial deficits in No-Go responding that mimicked the behavior of younger animals with prefrontal cortex lesions (Winocur, 1992), while aged rats (>22 months of age) shifted responding away from bigger, delayed reward more slowly than younger rats (3–6 months of age), while exhibiting impaired orbitofrontal coding of non-optimal delayed reward, suggesting altered representation of reward with aging (Roesch et al., 2012).

Dopaminergic deficiency has been observed in the frontal cortex of aged rats (>24 compared to <4 months of age) and correlated with impaired working memory in radial, T- and Morris water mazes, suggesting that age-related remodeling of mesocortical dopaminergic fibers may be an important feature of cognitive decline with aging (Luine et al., 1990; Mizoguchi et al., 2009; Allard et al., 2011). Further experiments have suggested that alterations in the regulation of the second messenger-dependent enzymes protein kinase A and protein kinase C in elderly rats may be an important factor in cognitive decline (Ramos et al., 2003; Brennan et al., 2009). Furthermore, delayed and decreased upregulation in gene expression has been observed for rats older than 20 months of age, in comparison to approximately 3 months of age, following pentylenetetrazole-induced seizure and stroke induced by reversible occlusion of the middle cerebral artery (Retchkiman et al., 1996; Buga et al., 2014). In addition, evidence suggests that variability in behavioral performance among individual animals may be explained, in part, by differences in age-related brain reserves, allowing varied baseline adaptive neuroplasticity (Freret et al., 2015). Thus, the rodent countermanding task may provide a new and interesting avenue to elucidate the underlying neural mechanisms of impaired behavioral control with aging.

Lengthening of RT following stop trial errors and shortening following correct go trials has been reported previously for rats performing the countermanding task (Beuk et al., 2014; Mayse et al., 2014). Of note, rats investigated presently were reported to exhibit these behaviors in subsequent vehicle sessions in comparison to 0.25 mg/kg amphetamine. Post-error RT lengthening observed at 7 and 12 months of age suggests that post-error RT lengthening in rats may be relatively consistent across much of the lifespan, consistent with findings in humans (Van de Laar et al., 2011; Hu et al., 2012). RT shortening following correct go trials was generally observed here, but failed to reach statistical significance, possibly due to the low number of subjects tested (Button et al., 2013).

Reduced total trials performed in 1-h countermanding sessions at 12, compared to 7 months of age was an unexpected finding. Humans commonly exhibit less physical exertion later in the lifespan and generally require more time to complete routine tasks (Owsley et al., 2002; Crombie et al., 2004). Variability in the number of trials completed within sessions of human countermanding testing during the lifespan were not reported, as these sessions typically ended following the completion of a specified number of trials as opposed to an amount of time (see Kramer et al., 1994; Williams et al., 1999; Bedard et al., 2002; Van de Laar et al., 2011). In one case, testing was conducted in 10-min blocks; however, a relationship between age and number of trials completed was not noted (Hu et al., 2012). Rats demonstrated decreased running-wheel and locomotor activity as well as deficits in complex motor behavior as early as 12 months of age, suggesting that older rats may be less physically able or engaged (Peng et al., 1980; Wallace et al., 1980; Willig et al., 1987; Van Waas and Soffié, 1996). Alternatively, the reduced performance may reflect motivational deficits related to impaired dopamine transmission (Salamone et al., 2015). Further investigation is required to more precisely determine the underlying nature of the reduction in trials performed by older animals.

Longer RT and reduced trials performed within sessions for rats at 12, compared to 7 months of age, may have resulted from the increased home cage feeding required to maintain weight growth as rats aged. Support comes from Baker et al. (2012), who noted differences in reinforcer devaluation depending upon alternative food restriction protocols. Unfortunately, ethical considerations make this hypothesis difficult to investigate, as continued growth of rats with age requires increased food availability. Thus, the effects of food restriction protocol may require some consideration in future rodent countermanding experiments. Changes in behavior observed presently may have also resulted from prolonged training and testing regimens. In contradiction to this hypothesis, rats exhibited lengthening of RT and a greater proportion of errors in later sessions, behaviors not usually linked with training effects. Nevertheless, it may be useful to investigate a separate cohort of older animals whereby initiation of training occurs later in the lifespan to rule out the possibility of training effects in the present observations. It would be of further merit to examine whether rats trained at an older age would acquire countermanding performance at a rate similar to younger animals.

Variability in rodent countermanding during the lifespan had yet to be directly considered, despite the growing exploration of rodent behavior in this task. Examination of the methodologies in previous reports suggests that the majority of testing was conducted on rats between 4 and 10 months of age. While substantial variability in control rat data is evident, inconsistencies in task design, subject strain and the lack of reporting age at the time of testing preclude identifying aging as an important factor (see Feola et al., 2000; Eagle and Robbins, 2003a,b; Van den Bergh et al., 2006; Eagle et al., 2007, 2009, 2011; Pattij et al., 2007, 2009; Robinson et al., 2008, 2009; Bari et al., 2009, 2011, 2014; Kirshenbaum et al., 2011; Bryden et al., 2012; Torregrossa et al., 2012; Bari and Robbins, 2013; Schmidt et al., 2013; Walker and Kissler, 2013; Mayse et al., 2014). Findings reported in Beuk et al. (2014) may suggest lifespan changes in performance when contrasting the behavior of the cohort examined presently following saline administration at approximately 13 months of age (RT ± SEM = 679 ± 30 ms; SSRT ± SEM = 186 ± 19 ms) to a separate cohort of treatment-free rats tested at approximately 6 months of age (RT ± SEM = 570 ± 17 ms; SSRT ± SEM = 157 ± 8 ms). Alternatively, data from Eagle and Robbins (2003b) and Eagle et al. (2008) do not appear to reveal substantially altered RT or SSRT for control rats tested at approximately 6 and 19 or 6 and 12 months of age, respectively.

Rats at 7 and 12 months of age exhibited remarkably consistent mean SSRT in the current experiment. Importantly, this implies that SSRT may be relatively comparable for adult rats in experiments of long durations, as is frequently the case in rodent countermanding. Future rodent experiments should report ages of test animals in order to further verify lifespan-related changes in countermanding performance. Notably, this study provides the first evidence that the age at which animals are tested is an important consideration in rodent countermanding investigation. The neural correlates of these behavioral changes merit further examination.

## Conclusion

Rats demonstrated changes in countermanding performance within the adult lifespan similar to those reported in adult humans, including RT lengthening and an increase in the proportion of errors. SSRT and post-error RT lengthening were relatively unchanged at the ages tested, suggesting that these behaviors are more resilient. Further experiments should examine more elderly rodents in the task to determine whether the observed effects of the present investigation are exacerbated and if inhibitory control and adaptive RT adjustment are substantially altered as rats become more elderly. This would fully characterize performance throughout the rodent lifespan. Together, these results suggest that rodent countermanding is an appropriate model to examine the neural correlates of aging related changes in behavioral control.

## Author Contributions

JB, RB, and MP contributed to the conception and design of this work, as well as the analysis and interpretation of data. JB contributed to the acquisition of data and the drafting and revision of this work. RB and MP contributed to manuscript revision.

## Conflict of Interest Statement

The authors declare that the research was conducted in the absence of any commercial or financial relationships that could be construed as a potential conflict of interest.
